# Homozygosity for the WRN Helicase-Inactivating Variant, R834C, does not confer a Werner syndrome clinical phenotype

**DOI:** 10.1038/srep44081

**Published:** 2017-03-09

**Authors:** Ashwini S. Kamath-Loeb, Diego G. Zavala-van Rankin, Jeny Flores-Morales, Mary J. Emond, Julia M. Sidorova, Alessandra Carnevale, Maria del Carmen Cárdenas-Cortés, Thomas H. Norwood, Raymond J. Monnat, Lawrence A. Loeb, Gabriela E. Mercado-Celis

**Affiliations:** 1 Departments of Pathology, University of Washington, 1959 NE Pacific St, Seattle, WA 98195, USA; 2 Biochemistry, University of Washington, 1959 NE Pacific St, Seattle, WA 98195, USA; 3 INMEGEN, National Institute of Genomic Medicine, Periferico Sur No.4809, Col. Arenal Tepepan, Del. Tlalpan Mèxico, D.F, C.P. 14610, Mexico; 4 Biostatistics, University of Washington, 1959 NE Pacific St, Seattle, WA 98195, USA; 5 National Institute of Medical Science and Nutrition Salvador Zubiran, Vasco de Quiroga 15, Colonia Sección XVI, Tlalpan C.P.14000, México D.F., Mexico; 6 Genome Sciences, University of Washington, 1959 NE Pacific St, Seattle, WA 98195, USA

## Abstract

Loss-of-function mutations in the *WRN* helicase gene cause Werner syndrome- a progeroid syndrome with an elevated risk of cancer and other age-associated diseases. Large numbers of single nucleotide polymorphisms have been identified in *WRN*. We report here the organismal, cellular, and molecular phenotypes of variant rs3087425 (c. 2500C > T) that results in an arginine to cysteine substitution at residue 834 (R834C) and up to 90% reduction of WRN helicase activity. This variant is present at a high (5%) frequency in Mexico, where we identified 153 heterozygous and three homozygous individuals among 3,130 genotyped subjects. Family studies of probands identified ten additional TT homozygotes. Biochemical analysis of WRN protein purified from TT lymphoblast cell lines confirmed that the R834C substitution strongly and selectively reduces WRN helicase, but not exonuclease activity. Replication track analyses showed reduced replication fork progression in some homozygous cells following DNA replication stress. Among the thirteen TT homozygotes, we identified a previously unreported and statistically significant gender bias in favor of males (p = 0.0016), but none of the clinical findings associated with Werner syndrome. Our results indicate that WRN helicase activity alone is not rate-limiting for the development of clinical WS.

Werner syndrome (WS) is a segmental progeroid and genomic instability disorder [refs 1,2 and references therein]. It is characterized by the premature onset of aging and an elevated incidence of cancer in individuals who have null mutations in both alleles of the WS gene, *WRN*^3^,^4^. Greater than 95% of clinically ascertained mutations in *WRN* are frameshift and nonsense mutations^5^,^6^,^7^,^8^, which predict truncated protein products. In all examined cases of cells derived from WS patients, however, neither the RNA transcript nor the protein is detectable^9^,^10^. Reported missense amino acid substitutions, present as compound heterozygous mutations in a small subset of WS patients^11^,^12^,^13^,^14^, lead to protein instability and loss- the functional equivalent of null alleles. Thus, the development of WS appears to require the loss of the WRN protein and both of its encoded catalytic activities.

In addition to pathogenic variants in *WRN*, large numbers of naturally occurring single nucleotide polymorphisms (SNPs) have been identified. While most that have been analyzed thus far do not significantly alter the biochemical activities of WRN, we, and others, have identified three missense variants that preferentially diminish helicase activity (refs 12,15, and Kamath-Loeb, A., unpublished) and one variant that selectively reduces exonuclease activity^16^. The best-characterized non-synonymous SNP that preferentially reduces DNA helicase activity is rs3087425 (c. 2500C > T). This polymorphism results in an arginine to cysteine substitution at amino acid 834 within the WRN helicase domain. Recombinant R834C-WRN protein isolated from mammalian cells in culture displays markedly diminished (40- to 50-fold) ATPase and helicase activities^15^, with retention of WRN exonuclease activity. Biochemical analysis of cells from one heterozygous (CT) individual revealed that helicase activity was reduced ~2-fold, suggesting that the variant allele may be non-functional^15^.

In order to quantify the frequency of the c. 2500C > T variant and identify potential phenotypes associated with it, we genotyped 3,600 individuals of diverse ethnicities and found that it is present in individuals of Spanish descent, but not of other European or African-American ancestry^15^. We show here that this variant is present at a high frequency in the Mexican population, and took advantage of this finding to determine whether selective reduction of WRN helicase activity contributes to premature aging, genomic instability, or other phenotypes. Our findings indicate that homozygous TT individuals who have close to 90% reduction of WRN helicase activity do not present signs or symptoms diagnostic of Werner syndrome in the Mexican population. In contrast to WS where there is a 1:1 ratio of male: female patients^17^, we observe a statistically significant gender bias in favor of males among homozygous TT individuals. This observation raises the possibility that WRN helicase activity may play a role in germ cell development and/or X-chromosome inactivation.

## Results and Discussion

The human *WRN* locus has a large number of coding and non-coding sequence variants. Among the 759 variants identified in the *WRN* coding region and splice junctions in the 1000 Genomes, Exome Sequencing Project, and the Exome Aggregation Consortium (ExAC) data, nearly all (97.6%) are single base substitutions^18^. The identification of prevalent non-synonymous *WRN* variants led quickly to studies aimed at identifying the association between specific variants and human heritable or acquired disease states. For example, the contribution of three common missense substitutions, V114I, L1074F and C1367R to potential age-associated conditions and cancer risk has been extensively analyzed in a number of populations^19^,^20^,^21^,^22^,^23^,^24^,^25^,^26^. In this study, we focused on a unique non-synonymous polymorphism, c.2500C > T (p. R834C) that we previously showed selectively reduces WRN DNA helicase, but not DNA exonuclease activity *in vitro*^15^. Among the 3,600 anonymous DNA samples from various ethnicities that we genotyped, we found that it is present in individuals of Spanish descent^15^. Here, we show that the variant is present at a high frequency in Mexico, which enabled us to evaluate the effect of WRN helicase deficiency on cellular, molecular, and clinical phenotypes in a cohort of R834C homozygous individuals.

### Distribution of WRN c. 2500C > T in Mexico

A large number of anonymous DNA samples from the Mexican HapMap collection^27^ were genotyped to determine the frequency of the *WRN* c. 2500C > T variant. Analyses of ~2,400 samples yielded a heterozygous allele frequency of 2%, with similar frequencies across both genders and in all examined states in Mexico (Table 1). Heterozygotes were observed in both indigenous and non-indigenous populations (Table 1); one TT homozygote was identified as well in this collection.

### Identification of WRN c. 2500C > T Heterozygous and Homozygous Individuals

Based on the above analyses, we recruited and genotyped 3,130 individuals from Mexico City. Individuals had to be at least 18 years old, born in Mexico, and have 1^st^ and 2^nd^ generation Mexican-born relatives to be included in the study. The study population age range was 18–104, and had a male: female ratio of 0.43:0.57 (Table 2a). Genomic DNA was isolated from blood samples and DNA integrity was verified by gel electrophoresis. TaqMan-based genotyping was carried out on coded duplicate samples and interpreted by two independent investigators. DNA samples identified as being heterozygous or homozygous by the TaqMan assay were subjected to Sanger sequencing to confirm the genotype. We identified 153 heterozygous individuals for a heterozygous (CT) allele frequency of 2.5% (Table 2a), very close to the 2% observed in the Mexican HapMap samples (Table 1). 253 *WRN* c. 2500C > T alleles are reported in the ExAC database, with nearly all (244 of 253, or 96.4%) of these in the Latino population where a total of 11,570 alleles were sequenced. The resulting heterozygous allele frequency of 2.1% is thus in close agreement with the frequency of 2.5% we identified in our Mexican study population (95% CI = [2.1 to 2.8%]). Based on this heterozygous allele frequency, we expected to identify 2 homozygous individuals among the ~3,000 individuals genotyped. We identified three homozygotes indicating that the major (C) and minor (T) alleles are in Hardy-Weinberg equilibrium (p = 0.45 for the HW exact test).

In order to determine whether the WRN R834C substitution confers an identifiable phenotype/s, we identified additional homozygous individuals in families of probands (heterozygous and homozygous) from our collection of 3,130 subjects. We visited 79 families residing in the greater Mexico City area to conduct histories and physical examinations of family members and to obtain blood samples for genotyping (under IRB guidelines). Genotype analyses (TaqMan PCR and direct sequencing) of these samples revealed an additional ten TT homozygous individuals (ages 18–61 yr; Table 2b). In all, thirteen homozygous individuals were identified in nine unrelated families (see Pedigree Charts, Supplementary Figs S1a–i). We also visited 17 control families (consisting of 71 members) to collect comparable control data. The ratio of male to female individuals in the family studies was 0.39:0.61.

### Expression and Enzymatic Activity of R843C-WRN Protein in TT Homozygous Cells

We had previously shown that R834C-WRN protein immuno-purified from transfected 293T cells had markedly reduced DNA helicase activity *in vitro*^15^. In order to determine if TT homozygous individuals also have reduced WRN helicase activity, we established EBV-immortalized cell lines of lymphocytes isolated from control and homozygous individuals from four independent families (Family #s 14, 19, 26, and 33). Early passage log phase cells were collected and lysed to quantify WRN protein levels and enzymatic activity as previously described^15^,^28^.

#### Protein levels

**Figure 1:**
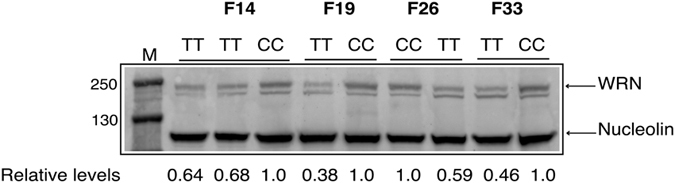
R834C-WRN is expressed in homozygous cells. Lysates (100 μg total protein) from control and TT homozygous cells from four pedigrees, F14, F19, F26, and F33, were electrophoresed through a 4–12% polyacrylamide gel. The proteins were blotted to a PVDF membrane, and probed with anti-WRN antibody and, simultaneously with anti-nucleolin antibody, to control for loading differences. The membrane was incubated with AlexaFluor 647-labeled anti-mouse antibody, and protein bands were visualized on an AlphaInnotech FluorChemQ Imaging Station. Band intensities were quantified using ImageJ software. CC: wild type controls; TT: R834C-WRN expressing cells; M: Molecular weight standards; numbers on the left indicate protein sizes in kDa.

100 μg of high salt protein lysates were electrophoresed through a 4–12% acrylamide gradient gel, blotted onto a PVDF membrane, and probed with a WRN-specific mouse monoclonal antibody. As seen in Fig. 1, when normalized to the loading control of nucleolin, R834C-WRN protein levels ranged from 40–70% of within-family control WRN levels. Thus, unlike Werner syndrome patients who lack detectable WRN protein, TT homozygous individuals express the variant protein, although at slightly reduced steady-state levels.

#### Enzymatic Activity

**Figure 2:**
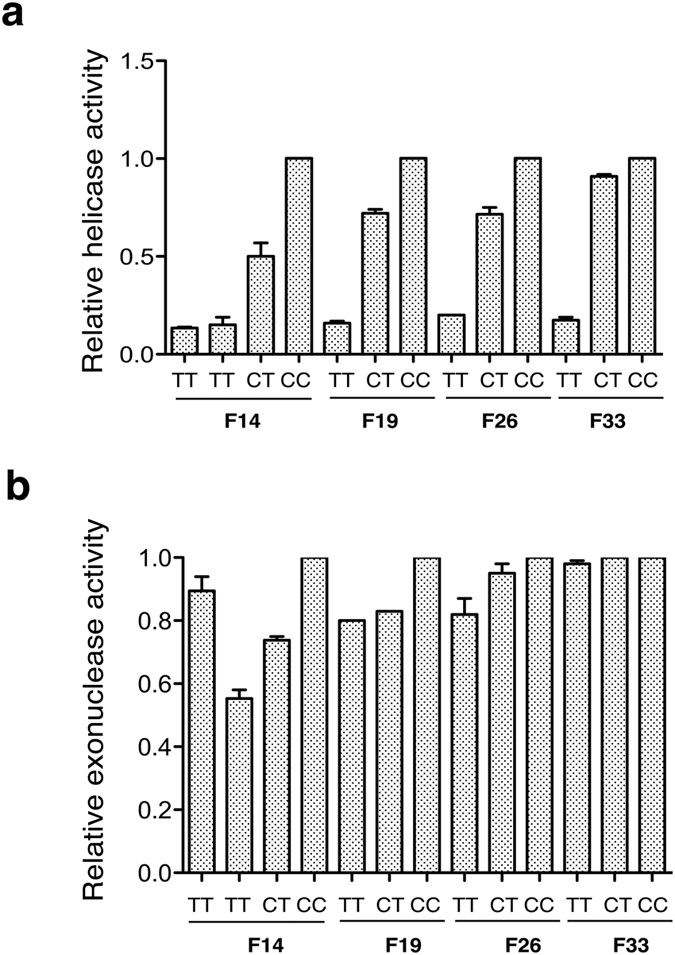
*WRN* c. 2500C > T (p. R834C) selectively reduces DNA helicase, but not DNA exonuclease, activity. Endogenous WRN in lysates (1 mg total protein) prepared from control (CC), heterozygous (CT), and homozygous (TT) cells from four pedigrees (F14, F19, F26, and F33) was affinity purified with a polyclonal anti-WRN antibody. Immunoprecipitates containing WRN were assayed for either DNA helicase (**a**) or DNA exonuclease (**b**) activity. Unwinding activity was measured using a 3′-end blocked 20/46 nt partial DNA duplex substrate, while exonuclease activity was assessed in the presence of ATPγS using the unblocked substrate; both substrates were 5′-end labeled using γ[^32^P]ATP and T4 polynucleotide kinase. Reactions were incubated at 37 °C for 20 min and aliquots were electrophoresed through native 12% (helicase) or denaturing 14% (exonuclease) polyacrylamide gels. Radioactivity in bands was visualized on a PhosphorImager, and band intensities were quantified using Image J software. Enzymatic activity in heterozygous and homozygous cells was normalized to that of within-pedigree controls.

WRN from control (CC), heterozygous (CT), and homozygous (TT) cell lysates was immunoprecipitated and assayed for WRN-associated DNA helicase and DNA exonuclease activities as previously described^15^,^28^. The specificity of the anti-WRN antibody for WRN was demonstrated by the lack of measurable enzymatic activity in immune precipitates from WRN minus cells^28^. The use of a DNA substrate containing a blocked 3′-end, or the inclusion of ATPγS, enabled us to examine, respectively, WRN helicase activity in the absence of nuclease activity, or exonucleolytic degradation in the absence of confounding DNA unwinding activity. Representative helicase and exonuclease activity gels are presented in Supplementary Fig. S2a,b, respectively, with quantified band intensities from independent experiments plotted in Fig. 2a,b. As shown, there is a 5- to 9-fold reduction of R834C-WRN helicase activity among the five homozygous cell lines compared to within-family control cells. In contrast, there is minimal, if any, decrease in WRN exonuclease activity in TT homozygous cells. Thus, as we had shown for purified recombinant R834C-WRN protein, WRN immuno-purified from TT homozygous cells exhibited selectively reduced DNA helicase, but not DNA exonuclease, activity. R834C-WRN purified from the lymphoblasts of homozygous TT individuals had between 10–20% of control WRN helicase activity, in contrast to the ~2% residual helicase activity we observed in recombinant R834C-WRN protein that was transiently over-expressed in 293T cells^15^.

### Molecular Phenotypes of TT Homozygous Cells

In order to determine whether TT homozygous cells exhibit molecular phenotypes characteristic of WS cells, we monitored DNA replication fork rates in response to hydroxyurea (HU)-mediated replication fork slowing. We previously used this quantifiable, sensitive, and mechanistically revealing assay to show that lowering WRN levels to ≤10% of normal by shRNA-mediated protein depletion renders cells sensitive to several DNA damaging agents, and increases the fraction of stalled replication forks^29^. Un-repaired stalled replication forks have the potential to lead to genomic rearrangements, deletions, and genetic instability as observed in WS patient cells^30^,^31^.

Actively replicating control [CC (n = 4)] and homozygous [TT (n = 5)] cells were incubated with ethynyldeoxyuridine (EdU) prior to the addition of HU, and then incubated with iododeoxyuridine (IdU) following removal of HU. Labeled DNA was then isolated, stretched, and immunostained on cover slips to visualize replicated DNA molecules. The lengths of fluorescent DNA tracks pre- and post-HU treatment were measured to determine whether HU alters DNA chain elongation rates in TT homozygous cells to a greater extent than in control cells.

**Figure 3:**
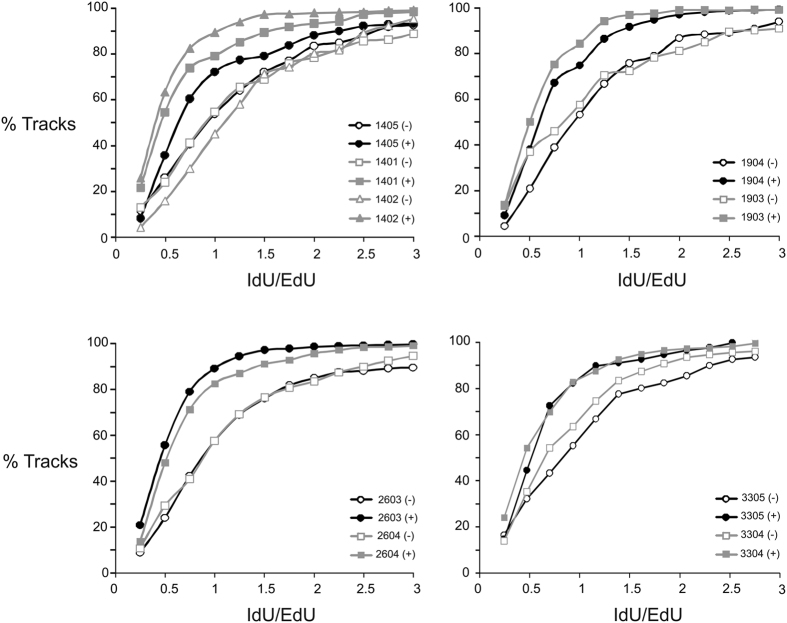
Some *WRN* c. 2500C > T homozygous cells display replication fork phenotypes. Replicating DNA in log phase control and TT homozygous LCLs were labeled with halogenated nucleosides, 5-ethynyldeoxyuridine (EdU) and iododeoxyuridine (IdU) before and after incubation with hydroxyurea (HU), respectively. Cells were lysed, and high molecular weight DNA was isolated and stretched on cover slips using pre-fabricated microchannels. EdU and IdU tracks were stained with fluorescent antibodies and visualized by fluorescent microscopy (Zeiss Axiovert microscope). Track lengths, pre- and post- HU treatment, were measured using AxioVision software. Cumulative ratios of 2^nd^ to 1^st^ labeled segments in two-segment tracks (n = 100–200/sample) were calculated and graphed. A decrease in the rate of DNA chain elongation is manifested as a left shift (decreased IdU/EdU ratios). Controls (black lines): 1405, 1904, 2603, and 3305; Homozygous (grey lines): 1401, 1402, 1903, 2604, and 3304. Open symbols: (−) HU; closed symbols: (+) HU.

Two TT homozygous cell lines, 1401 and 1402, displayed shorter DNA tracks post-HU compared to the within-family control, as reflected in the left shift of IdU/EdU track ratios in Fig. 3. This difference between the homozygous and control cells was highly significant (p values = 5.6 × 10^−4^ and 1.7 × 10^−7^ for 1401 and 1402, respectively; Wilcoxon test). Differences in the IdU/EdU ratio were observed only in response to HU, indicating that TT homozygous cells display this fork phenotype only under conditions of replication stress. In contrast to cell lines 1401 and 1402, track lengths in the remaining TT homozygous lines were either shortened to similar extents (1903 and 3304), or even lengthened (2604), compared to respective family controls after exposure to HU. Of note, among the five analyzed homozygous lines, 1401 and 1402, which showed the greatest reduction in replication track lengths, also showed the greatest reduction of WRN helicase activity (Fig. 2).

The role of WRN helicase activity to cellular phenotypes has been well-documented^29^,^32^,^33^,^34^. For example, Sidorova *et al*.^29^,^33^ depleted WRN from cells in culture and found that WRN-depletion revealed replication fork phenotypes similar to that exhibited by WRN-null WS patient cells when challenged with DNA damaging agents. WRN-depleted cells that exhibit replication phenotypes have ≤10% of normal protein levels and enzymatic activity, and WS patient cells lack both detectable WRN protein and biochemical activity. *WRN* TT homozygous cells, in contrast, retain between 10–20% of WRN helicase activity. This may indicate a threshold of ~10% or less of native WRN helicase activity to sensitize cells to DNA damaging agents and to reveal WRN helicase-dependent cellular phenotypes. Some investigators have proposed that WRN serves a non-enzymatic function such that levels of WRN, even those of inactive proteins, determine cellular phenotypes^35^,^36^. If this were the case, TT homozygous cells would not be expected to exhibit dramatic phenotypes since R834C-WRN protein is expressed at levels that are 40–70% of normal.

### Phenotypes of WRN c. 2500C > T Homozygous Individuals

We used a combination of in-house history and physical examinations, as well as examinations by an internist and, in some instances, by a dermatologist and an ophthalmologist to determine whether TT homozygotes present phenotypes associated with Werner syndrome (Supplementary Tables S1 and S2). We ascertained WS ‘cardinal signs’ and ‘other signs and symptoms’ (see Methods for details of these features) using criteria defined by the International Registry of Werner syndrome to identify individuals with definite, probable, or possible WS^2^,^17^,^37^.

We observed some features of WS among TT homozygous individuals, e.g., grey hair, early onset hair loss, short stature, thin limbs, and flat feet, but these findings were neither consistent nor significantly more frequent among TT homozygotes than among other *WRN* genotypes (Table 3). For example, only individual 1402 had short stature and thin limbs, only individuals 6712 and 7502 had early-onset hair loss, while individuals 7309 and 7508 displayed none of these features. Two cardinal signs of WS, early onset bilateral cataracts and reduced fertility were absent in all TT homozygotes. Five of the 13 homozygotes are parents in the 4^th^ and 5^th^ decade of their life, the upper expected lifespan for WS patients, and showed no collective signs suggestive of WS up to age 61. WS patients also have an increased incidence of cancers including thyroid carcinomas, meningiomas, and soft tissue sarcomas^37^. No incidence of cancer was observed in the thirteen TT homozygotes, nor was there evidence of chromosomal aberrations in their cells (data not shown). The absence of cardinal signs of WS (bilateral cataracts, characteristic skin changes) in TT homozygotes does not support a clinical diagnosis of WS. Further, “additional signs of WS” (not cardinal), which were present in some TT homozygotes, were not present at significantly elevated frequencies compared to the CC or CT population controls (p > 0.05 for Fisher’s exact test comparison of these trait frequencies across the 3 genotypes; Supplementary Table S1). The strongest significance level for difference in frequency of additional signs of WS occurred for diabetes mellitus, present in 4/13 TT (31%), in 24/238 CT (10%) and in 36/259 CC (14%) individuals, although this was not statistically significant (p = 0.06 for a two-tailed test, and p = 0.03 for a one-tailed test for elevated frequency, which is not significant after Bonferroni correction for multiple-testing). By far the most significant difference we observed was in the subjective assessment of ‘older than actual age’; however, this feature was also not statistically significant after applying correction for multiple testing.

In contrast to *WRN*, several missense mutations have been identified in the related RecQ helicase, *BLM*, which result in Bloom syndrome- another recessive genomic instability and cancer predisposition disorder like Werner syndrome. These missense-mutant proteins lack BLM helicase activity^38^,^39^ and led us to expect that WRN mutant proteins lacking helicase activity might also lead to acquired diseases associated with WS. However, we did not identify WS clinical findings or disease phenotypes in individuals homozygous for the R834C (c. 2500C > T) vvariant in our Mexican population-based analysis. These results, together with the replication fork data (see above and refs 33 and 40) and our analysis of phenotypes of cells expressing WRN exonuclease-null, helicase-null, or double missense mutant WRN proteins^34^, all argue that WRN helicase activity alone is not rate limiting for WS pathogenesis, and that clinical WS likely results only when both WRN helicase and exonuclease activities are lacking in patient cells.

### Additional Phenotypes of WRN c. 2500C > T Homozygous Individuals

Despite the lack of clinical findings of WS among TT homozygotes, we found that poor visual acuity was more common among male TT homozygotes than among male heterozygotes and controls (Tables 3 and 4). All 11 (100%) male TT homozygotes we examined had significantly reduced visual acuity compared to 54 of 92 (59%) CC males and 53 of 97 (55%) CT males; however, the difference in frequency was not statistically significant (Supplementary Table S1). None of the TT homozygotes had early onset bilateral cataracts, a common finding in WS patients, to explain their impaired visual acuity.

**Figure 4:**
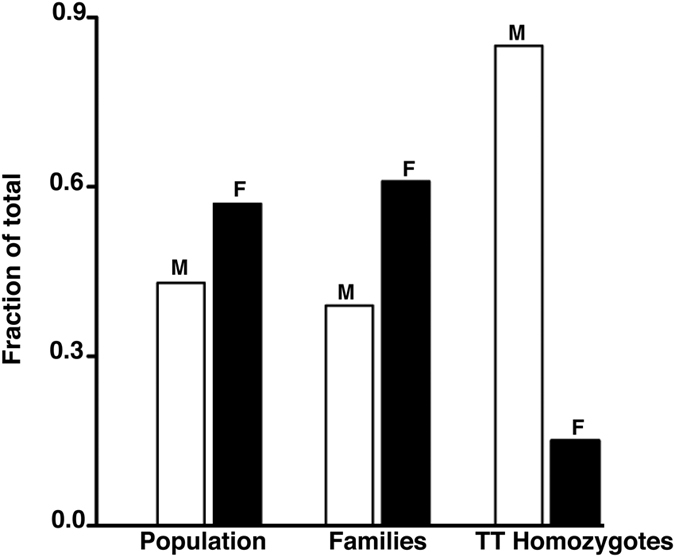
Gender distortion among *WRN* c. 2500C > T homozygotes. The fraction of male (M; open bars) and female (F, filled bars) participants in our study population of 3,130 individuals was 0.43 and 0.57, respectively, and that within the 513 family members was 0.39 and 0.61, respectively. In contrast, the ratio of male: female individuals among the TT homozygotes was skewed at 0.85:0.15.

Perhaps, the most unexpected finding in this cohort was a statistically significant skewed gender distribution of TT homozygotes (Tables 3 and 4). Eleven of thirteen homozygotes were male, yielding a male: female ratio of 0.85:0.15 that is markedly different from the male: female ratio of 0.39:0.61 in individuals genotyped from the 96 families, the 0.37:0.63 ratio among genotyped members of the nine families with one or more TT homozygotes (Fig. 4), or the ~0.5:0.5 (or 1:1) ratio observed in WS patients^17^ and in the population of Mexico (Mexico Demographics Profile 2016). The proportions of subjects by genotype and gender are 0.46 (92/200) CC, 0.49 (97/200) CT, and 0.06 (11/200) TT males *versus* 0.54 (168/313) CC, 0.46 (143/313) CT, and 0.006 (2/313) TT females. The gender ratio skewing in TT carriers was statistically significant both before and after correction for multiple testing (p value = 0.0016, by 2 × 3 Fisher’s 2-tailed tests; p value = 0.025 after correction; Supplementary Table S1). This distorted gender ratio in TT homozygotes is not due to age differences between males and females- age distributions by decade for the two sexes were almost exactly the same. Furthermore, there was no relationship between genotype and age within either sex, not for any of the three genotypes for males and not for CT *versus* CC for females. That is, among males, the proportions of TT, CT, and CC persons were equivalent across all ages as it was for females of CC and CT genotypes (Supplementary Tables S1 and S2).

The gender imbalance in favor of males in TT homozygotes appears to be constitutional, not acquired, and raises the interesting possibility that WRN helicase may influence gender ratio by virtue of a role in germ cell development or in X-chromosome inactivation. Our data are reminiscent of other reports of gender skewing caused by mutations in other DNA repair and genome stability maintenance proteins such as BRCA1 and FANCC. BRCA1 interacts with WRN and plays a role in X-chromosome inactivation^41^,^42^. Carriers of *BRCA1* mutations show gender skewing, though in favor of females as opposed to males^43^,^44^. Additionally, mice mutant for the Fanconi anemia Fancc protein, which plays a role in DNA cross-link repair and germ cell development^45^,^46^ also display strong skewing of gender in favor of males (D.W. Clapp, unpublished data). These observations, and our results in *WRN* TT homozygotes, highlight potentially novel roles for different DNA repair proteins in germ cell development.

Our identification of the high heterozygous frequency of the non-synonymous *WRN* polymorphism, c. 2500C > T (p. R834C) in Mexico allowed us to determine whether selective inactivation of WRN helicase activity confers cellular phenotypes, clinical findings or disease risks characteristic of Werner syndrome in TT homozygotes. While we confirmed that TT homozygotes do indeed have marked and selective reduction of WRN DNA helicase activity, they lack other highly penetrant clinical findings observed in WS patients. TT homozygotes instead, revealed previously unreported findings, distinct from classical WS features, of gender distortion in favor of males (Table 4), a higher frequency of diabetes mellitus and poor visual acuity. The latter two findings were not statistically significant after adjusting for multiple-testing, though we note that the statistical power of the analysis was low due both to the rarity of the TT genotype and the large number of tests performed. Independent follow-up study will be needed to further confirm the most interesting of our findings, and to explore potential mechanisms by which WRN helicase activity may determine germ cell development and/or gender parity at birth.

## Materials and Methods

### Recruitment and Sample Collection

Individuals for the study were recruited from the National Autonomous University of Mexico (UNAM), the National Institute of Medical Science and Nutrition Salvador Zubiran (INNCMSZ), and the National Institute of Genomic Medicine (INMEGEN). Inclusion criteria required individuals to be adults (>18 years old), and have both parents and grandparents of Mexican descent. All studies involving human subjects, human specimens, and human data were approved by the Institutional Review Boards at the National Institute of Genomic Medicine in Mexico, at the University of Washington in Seattle, Washington, and at all other participating institutions. Human subject activities were performed in accordance with the regulatory requirements laid down in the U.S. Code of Federal Regulations, Title 45 Department of Health and Human Services Part 46, Protection of Human Subjects. Informed consent was obtained from all participants included in the study.

### Genotype Analysis

Blood samples (18 ml) were collected from 3,130 recruited individuals, and sent to the National Institute of Genomic Medicine for DNA isolation and for *WRN* rs3087425 (c. 2500C > T) variant genotyping. Genomic DNA was extracted from buffy coat using Gentra Puregene Blood Kit (Qiagen) according to the manufacturer’s recommendations. DNA concentrations were determined by NanoDrop measurements (Thermo Scientific), and DNA integrity was assessed by electrophoresis through 1% agarose gels prior to genotyping using TaqMan SNP Genotyping Assays. The assay-on-demand for *WRN* rs3087425 was obtained from Applied Biosystems. Real-time PCR was performed with 10 ng of genomic DNA template in 10 μl reaction mixtures containing 5 μl of TaqMan universal master mix (Applied Biosystems), 0.25 μl of Custom TaqMan^®^ SNP Genotyping Assays, and 3 μl of sample DNA. Thermocycling was performed on the ViiA™ 7 Real-Time PCR System (Applied Biosystems) using recommended cycling conditions- 95 °C for 10 min, followed by 40 cycles of denaturation at 95 °C for 15 sec and annealing/extension at 60 °C for 1 min. Each assay was carried out in duplicate, and analyses were performed using SDS software, version 2.0. The genotype of each sample was manually verified by visual examination of PCR curves.

### Family Studies

Individuals positive for the *WRN* c. 2500C > T variant (as heterozygous or homozygous carriers) and respective family members were invited to participate in a family study. As controls, we included 17 families whose members did not carry the variant allele. Informed consent was obtained from all individuals prior to conducting interviews and medical examinations. A total of 96 families, comprising 513 members, were recruited in this phase of the study.

Demographic data including gender, age, and degree of kinship to the index case were collected for index cases and family members. Individuals were evaluated for a large number of features, with a focus on phenotypes associated with Werner syndrome (Supplementary Tables S1 and S2). We used criteria established by the International Registry of Werner syndrome^17^,^37^ to evaluate both cardinal signs (bilateral cataracts, dermatological pathology, short stature, and premature graying and/or thinning of scalp hair) and additional signs and symptoms (diabetes mellitus, osteoporosis, soft tissue calcification, neoplasia, voice changes, and flat feet) associated with WS and determine whether *WRN* TT homozygotes met these criteria for diagnosis of WS. Further, we used Fisher’s exact test to assess significance of differences in frequency across genotypes for each of these 14 traits as well as for sex and appearance older-than-actual age. This results in a total of 16 formal tests for which both nominal and Bonferroni-corrected p-values are provided (Supplementary Table S1). TT homozygotes were further examined by an internist, and in some cases, by an ophthalmologist and a dermatologist at the National Institute of Medical Science and Nutrition Salvador Zubiran (INNCMSZ) to score key WS clinical findings, e.g., slit lamp evaluation to detect early-onset ocular cataracts. All of the samples and information used in the study were coded, and patient confidentiality was preserved according to the guidelines for studies of human subjects.

### Establishment of Lymphoblast Lines

Blood samples were drawn from control, heterozygous (CT), and homozygous (TT) individuals into 10 ml vacutainer tubes containing 1 ml ACD-A (anticoagulant citrate dextrose solution A; VWR) and express mailed from Mexico City to Seattle at room temperature. Following receipt, samples were centrifuged at 300 × g for 20 min to remove plasma and recover the buffy coat containing lymphocytes. Lymphocytes were further separated by centrifugation through Ficoll-Paque PLUS separation medium (Amersham Biosciences), and then washed twice in RPMI1640 medium lacking serum. Lymphoblastoid cell lines were generated from purified lymphocytes by infection with Epstein-Barr virus at 37 °C under 5% CO_2_ as previously described^47^. The resultant lymphoblast cell lines (LCLs) were expanded and propagated at 37 °C under 5% CO_2_ in RPMI1640 medium supplemented with 15% heat-inactivated fetal bovine serum.

### Immunoblot Analyses

Log phase LCLs (~10^6^ cells/ml) were harvested, washed 1× in PBS, and flash frozen in liquid N_2_. Cell pellets were lysed in high salt buffer as described previously^28^, and protein concentrations were measured by the Bradford assay. Approximately 100 μg of total protein were electrophoresed through a NUPAGE 4–12% Bis-Tris polyacrylamide gradient gel (Life Technologies) following the manufacturer’s guidelines. The gel was then transferred overnight to a PVDF membrane (Immobilon-FL, Millipore) using Tris-glycine-methanol transfer buffer. Non-specific membrane interactions were minimized by incubation in a 5% milk solution prepared in TBS-T (Tris-buffered saline, pH 7.5 + 0.05% Tween 20). The membrane was incubated overnight at 4 °C with a WRN- specific mouse monoclonal antibody (clone 195C; Sigma; 1:1000)^40^ and simultaneously with mouse anti-nucleolin antibody (Invitrogen; 1:1000) to control for loading differences. The membrane was incubated with AlexaFluor 647 anti-mouse antibody (Invitrogen; 1:1000) for 1 hr at room temperature, washed three times in TBS-T, and then imaged on an AlphaInnotech FluorChemQ Imaging Station. Band intensities were quantified using ImageJ software.

### Immune Precipitation and Enzymatic Assays

Endogenous WRN in protein lysates (1.0 mg total protein) was precipitated with a rabbit polyclonal antibody (9130 J) and heat-inactivated *S. aureus* cells (Pansorbin, CalBiochem), as described previously^15^,^28^. Incubations were carried out in IP buffer (20 mM Tris-HCl, pH 8.0, 150 mM NaCl, 25% glycerol, and detergents-0.5% Igepal, 0.05% sodium deoxycholate, 0.005% SDS) for 60 min at 4 °C. Immunoprecipitates were washed 3× with 0.5 ml IP buffer, and resuspended in buffer containing 25 mM Tris-HCl, pH 8.0, 0.5 mM EDTA, 1 mM DTT, 0.05% Igepal, and 25% glycerol.

Helicase activity in resuspended immune precipitates was monitored by the displacement of a 3′-blocked 20-mer hybridized to a complementary 46-mer DNA oligonucleotide at 37 °C for 20 min, as previously described^48^,^49^. Exonuclease activity was measured with an unblocked 20-mer/46-mer partial duplex DNA substrate in the presence of the non-hydrolyzable ATP analog, ATPγS^16^. In both cases, the 20-mer was labeled at the 5′-end using γ[^32^P]ATP and T4 polynucleotide kinase. Aliquots of the helicase and exonuclease reactions were electrophoresed through 12% native or 14% denaturing polyacrylamide gels, respectively. The displaced 20-mer and its degradation products were visualized on a PhosphorImager (GE Health Care), and quantified using ImageJ software.

### Replication Track Analysis (maRTA)

Replication fork progression in the absence or presence of hydroxyurea (HU) was monitored as described by Sidorova *et al*.^50^. First, log phase CC and TT homozygous LCLs were incubated with the halogenated nucleoside, 5-ethynyldeoxyuridine (EdU; 10 μM) for 45 min. HU (final concentration 2 mM) was subsequently added and incubation was carried out for an additional 5 hrs. Thereafter, cells were washed once with PBS to remove EdU and HU, and incubated in medium containing a second halogenated nucleoside analog, iododeoxyuridine (IdU; 50 μM) for 45 min. Labeled cells were again washed and embedded in agarose plugs using a solution of 2% low melt agarose. Cells in agarose plugs were lysed overnight at 55 °C in buffer containing Proteinase K and detergents. The plugs were then melted at 75 °C and incubated overnight at 42 °C with β-agarase to release high molecular weight DNA.

DNA was stretched on silanized cover slips using pre-fabricated PDMS microchannels. EdU-containing DNA was detected by biotin Click-It chemistry and staining with Texas red neutravidin^51^. IdU was detected by incubation with mouse anti-IdU antibody followed by incubation with Alexa 488 anti-mouse antibody. The cover slips were mounted on glass slides, and DNA tracks (300–400 single and double-labeled tracks/sample) were visualized and imaged using a confocal immunofluorescent Zeiss Axiovert microscope. Lengths of replication tracks pre- (red-labeled) and post- (green-labeled) HU treatment were measured using AxioVision software^50^. Cumulative distributions of IdU/EdU ratios ± HU were calculated and plotted.

## Additional Information

**How to cite this article:** Kamath-Loeb, A. S. *et al*. Homozygosity for the WRN Helicase-Inactivating Variant, R834C, does not confer a Werner syndrome clinical phenotype. *Sci. Rep.* **7**, 44081; doi: 10.1038/srep44081 (2017).

**Publisher's note:** Springer Nature remains neutral with regard to jurisdictional claims in published maps and institutional affiliations.

## Supplementary Material

Supplementary Information

Supplementary Table 1

Supplementary Table 2

## Figures and Tables

**Table 1:** Distribution of the major ‘C’ and minor ‘T’ alleles of *WRN* rs3087425 among females and males within the indicated states (top) and indigenous populations (bottom) of Mexico.

State	Gender	CC	CT	TT
Guanajuato	Female	81	5	—
	Male	86	3	—
DDD Durango	Female	86	1	—
	Male	85	6	—
Oaxaca	Female	87	5	—
	Male	84	5	—
Veracruz	Female	84	5	—
	Male	82	8	—
Yucatan	Female	87	4	—
	Male	78	3	—
Campeche	Female	89	3	—
	Male	86	6	—
Tamaulipas	Female	89	3	—
	Male	89	3	—
Guerrero	Female	88	4	1
	Male	87	5	—
Sonora	Female	91	1	—
	Male	89	3	—
Zacatecas	Female	84	8	—
	Male	88	4	—
**Indigenous Community**				
Maya	Female	87	4	—
	Male	88	4	—
Mixtecos	Female	87	2	—
	Male	80	2	—
Tepehuanes	Female	91	0	—
	Male	85	0	—
Zapotecos	Female	11	1	—
	Male	14	0	—

**Table 2:** Characteristics of Individuals Genotyped in the Population **(a)** and in Families **(b)**.

Characteristics	Subjects	%
**a**		
**Enrolled**	3130	
Gender		
Female	1772	57
Male	1358	43
Age		
Median (SD)	25.77 (14.39)	
Min-Max	18–104	
Genotype		
TT	3	0.1
CT	153	4.9
CC	2974	95
Alleles		
T	159	2.54
C	6101	97.46
**b**		
**Enrolled**		
Gender		
Female	313	61
Male	200	39
Age		
Median (SD)	43.14 (18.9)	
Min-Max	18–96	
Genotype		
TT	13	2.5
CT	240	46.8
CC	260	50.7
Alleles		
T	266	25.93
C	760	74.07

**Table 3:** Clinical Characteristics of R834C-WRN Homozygotes.

ID	Kinship	Age	Gender	Short Stature^1^	Grey Hair^2^	Thin Hair	Ame-tropia^2^	DM^2^	High BP^3^	Other Characteristics	
1401	Index	19	M	No	No	No	18	No	No	Ectomorphic somatotype; late pubarche	
1402	Sister	18	F	Yes	No	No	No	No	No	Thin Limbs	
1903	Parent	56	M	No	50	No	54	No	No	Late pubarche	
2604	Parent	57	M	No	No	No	40	No	Yes	Hypermelanosis	
3104	Uncle	61	M	No	40	No	40	No	No	Hair loss	
3304	Parent	46	M	No	30	No	23	33	No	Hypermelanosis	
6701	Index	20	M	No	18	No	18	No	Yes	Older appearance^4^; flat feet^5^	
6712	Brother	25	M	No	No	No	24	No	No	Older appearance; early onset hair loss^6^; flat feet	
6801	Index	22	M	No	No	No	20	No	No	Older appearance; nail deformity; cold fingers; flat feet	
7303	Parent	42	M	No	No	Yes	29	32	Yes	Hypermelanosis; nail deformity; cold fingers; dyslipidemia	
7309	Uncle	39	M	No	35	Yes	39	29	No		
7502	Parent	58	M	No	54	Yes	55	58	Yes	Early onset hair loss; atrophic skin; bird-like facies	
7508	Aunt	59	F	No	48	No	No	No	No		

^1^Stature was based on mid-parental height and comparison to growth charts of the Mexican population.

^2^Numbers indicate age of appearance of grey hair, diagnosis of ametropia (visual acuity), or onset of diabetes mellitus (DM).

^3^BP: blood pressure.

^4^Description of older appearance was based on comparison with family members and identical aged. individuals in the Mexican population.

^5^Flat feet diagnosed at age 3 yr in all three individuals.

^6^Early onset hair loss is defined as hair loss before the age of 30.

**Table 4:** Salient Features of R834C-WRN Homozygotes.

ID	Gender	Ametropia	%Helicase Activity^1^	Replication Phenotype^2^
1401	M	Yes	13	1.6
1402	F	No	11	2.1
1903	M	Yes	15	1.1
2604	M	Yes	20	0.54
3104	M	Yes	ND	ND
3304	M	Yes	16	0.94
6701	M	Yes	ND	ND
6712	M	Yes	ND	ND
6801	M	Yes	ND	ND
7303	M	Yes	ND	ND
7309	M	Yes	ND	ND
7502	M	Yes	ND	ND
7508	F	No	ND	ND

^1^Normalized to within-pedigree wild type control activity, which is set at 100%.

^2^Numbers refer to the ratio of mean IdU/EdU replication track length ratios in the presence or absence of HU in homozygous cells compared to within-pedigree wild type control cells.
