# Supplementary material for: Homozygosity for the WRN Helicase-Inactivating Variant, R834C, does not confer a Werner syndrome clinical phenotype
Source: Sci Rep. 2017 Mar 9;7:44081. doi: 10.1038/srep44081 (PMC5343477; doi:10.1038/srep44081)
Supplement: Supplementary Information [file srep44081-s1.pdf]

Supplementary Information

Homozygosity for the WRN Helicase-Inactivating Variant, R834C, does not confer a Werner syndrome clinical phenotype

Ashwini S. Kamath-Loeb, Diego G. Zavala-van Rankin, Jeny Flores-Morales, Mary J. Emond, Julia M. Sidorova, Alessandra Carnevale, Maria del Carmen Cárdenas-Cortés, Thomas H. Norwood, Raymond J. Monnat Jr, Lawrence A. Loeb and Gabriela E. Mercado-Celis.

Legends to Supplementary Figures:

Supplementary Figs. S1a-i: Pedigree charts of families with TT homozygotes. Control (CC; open black squares/circles), heterozygous (CT; half-filled squares/circles), and homozygous (TT; filled squares/circles) participants in our studies are numbered and indicated in each chart. The arrow indicates the proband in each family. Grey squares and circles represent non-genotyped family members. Diamonds represent individuals of unknown gender; numbers within shapes indicate number of children of that particular gender; triangle represents spontaneous abortion; and vertical line ending in a horizontal line indicates no children.

Supplementary Fig. S2: *WRN* c. 2500C>T (p. R834C) selectively reduces DNA helicase, but not DNA exonuclease, activity. Endogenous WRN in control (CC), heterozygous (CT), and homozygous (TT) cells from pedigrees F14, F19, F26, and F33 was affinity purified and assayed for DNA helicase (a) or DNA exonuclease (b) activity as described in 'Materials and Methods', and in the legend to Fig. 2. Representative gels used for quantitative analyses (Fig. 2) are shown. Δ: heat-denatured substrate; S: substrate (-) enzyme; rWRN: recombinant WRN protein.

Family 14

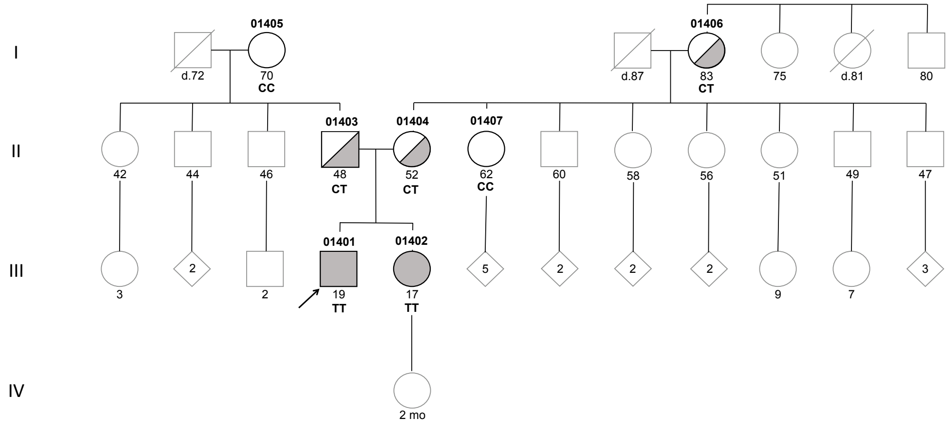

Supplementary Fig. S1a

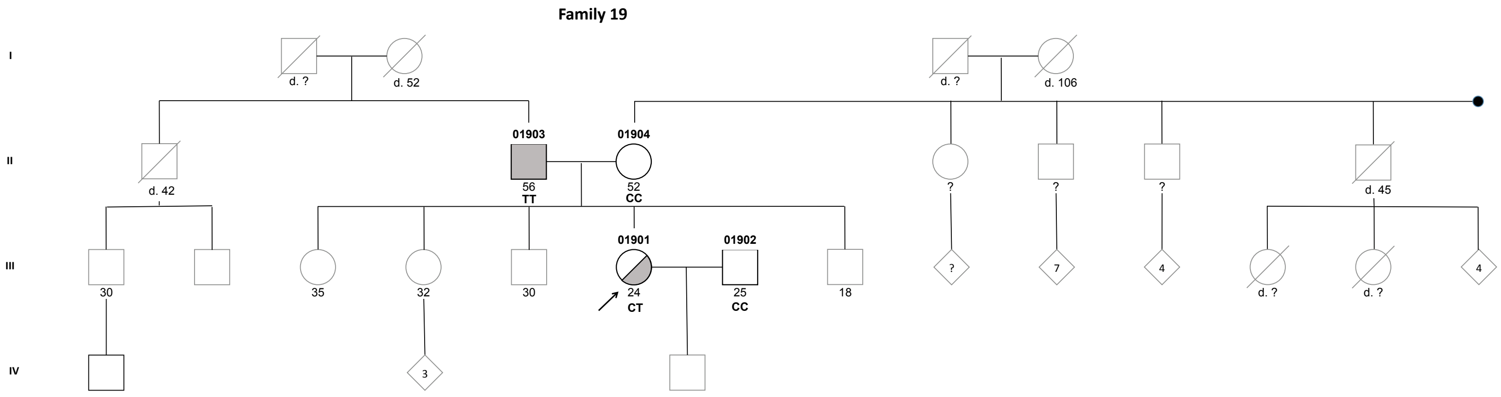

Supplementary Fig. S1b

Family 26

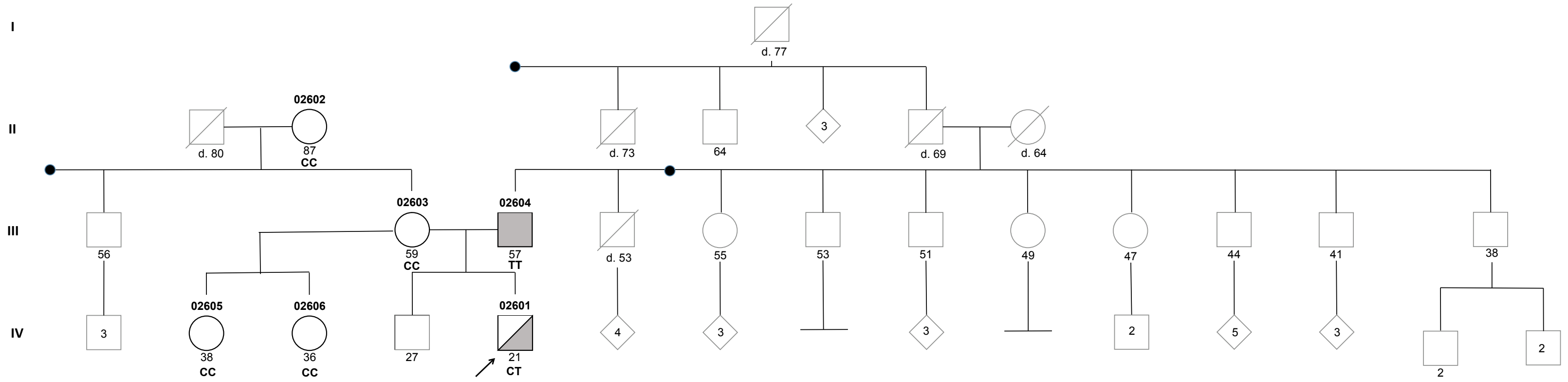

Supplementary Fig. S1c

Family 31

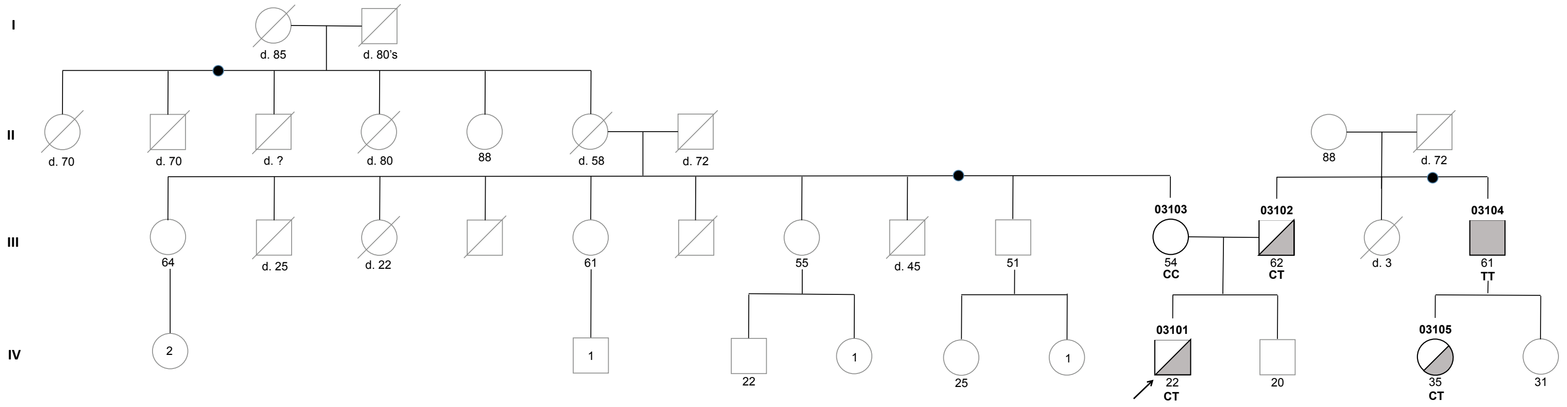

Supplementary Fig. S1d

Family 33

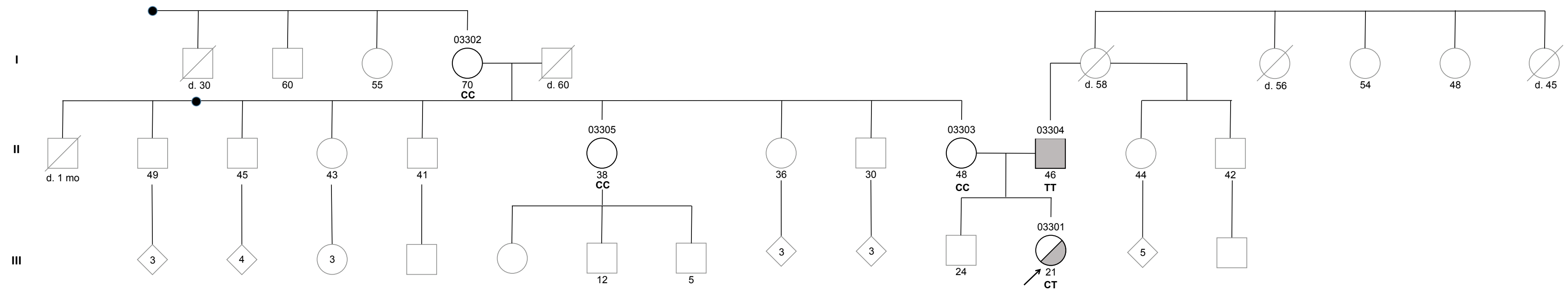

Supplementary Fig. S1e

Family 67

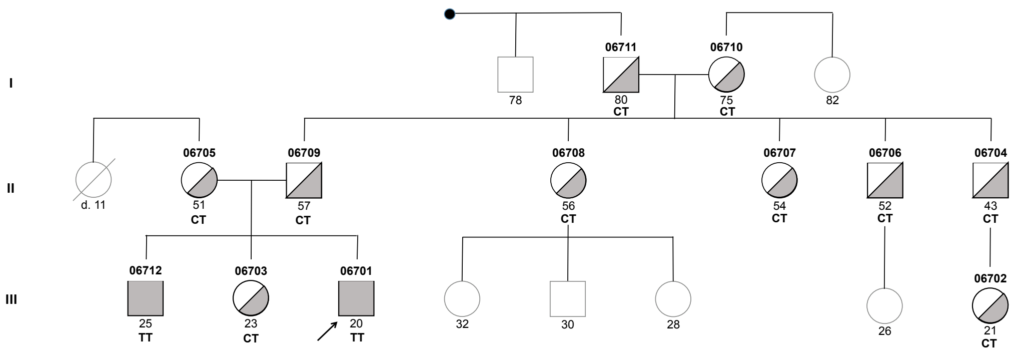

Supplementary Fig. S1f

Family 68

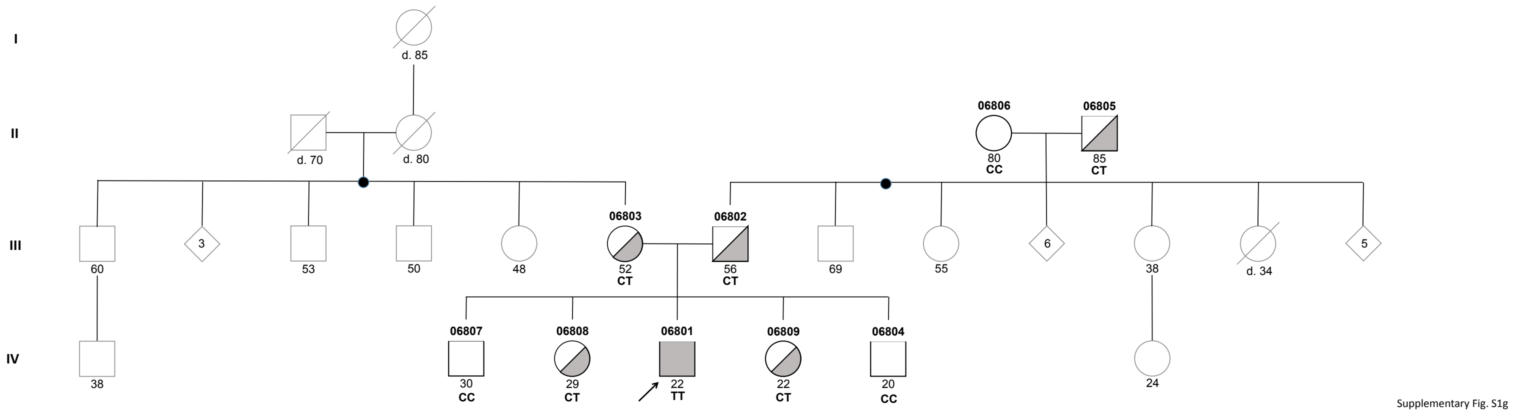

Supplementary Fig. S1g

Family 73

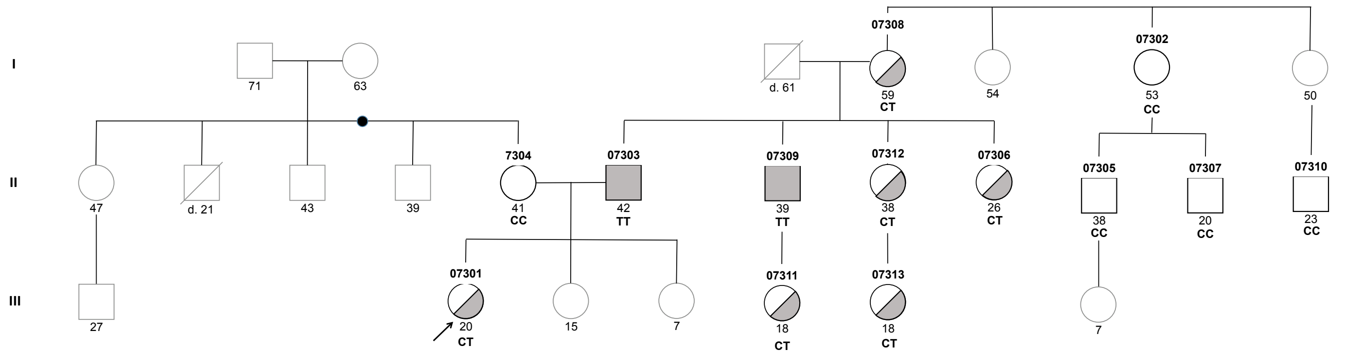

Supplementary Fig. S1h

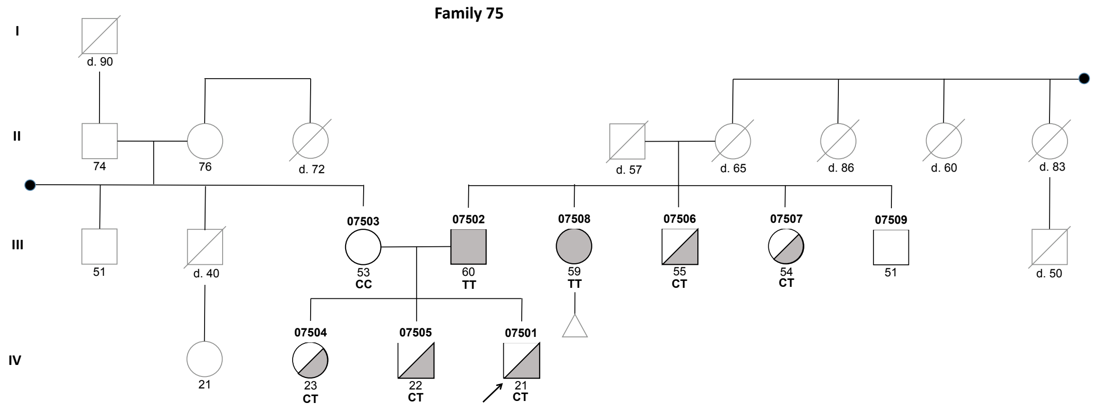

Supplementary Fig. S1i

a. Helicase

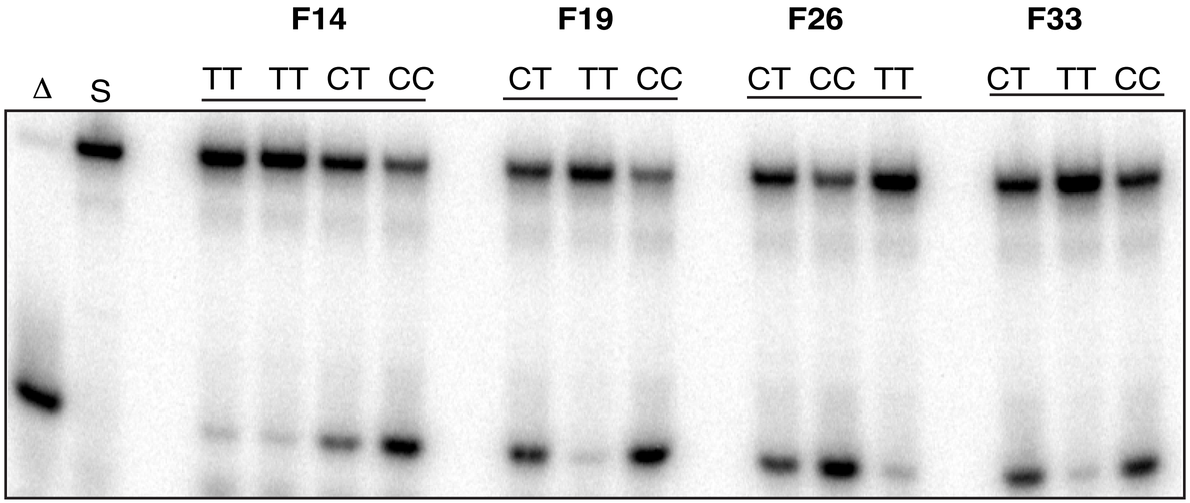

b. Exonuclease

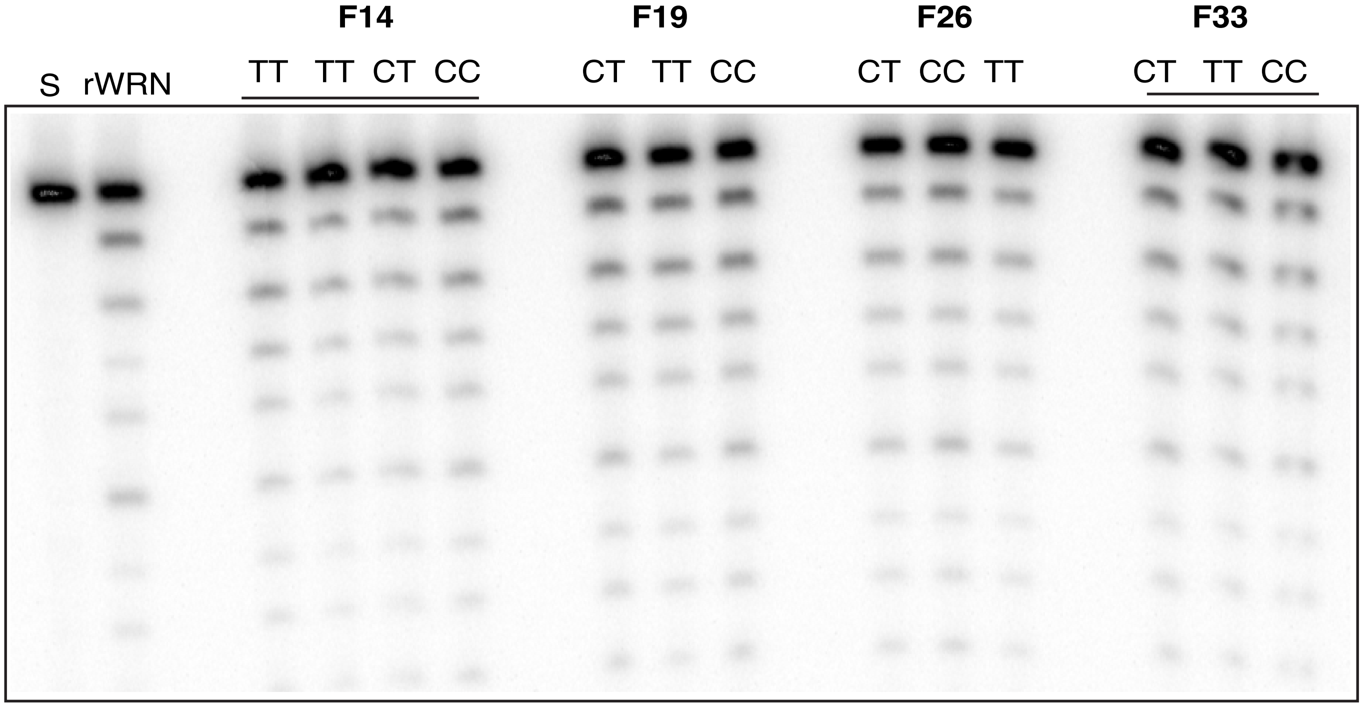

Supplementary Fig. S2
